# Neuro-Coagulopathy: Blood Coagulation Factors in Central Nervous System Diseases

**DOI:** 10.3390/ijms18102128

**Published:** 2017-10-12

**Authors:** Ciro De Luca, Assunta Virtuoso, Nicola Maggio, Michele Papa

**Affiliations:** 1 Laboratory of Neuronal Networks, University of Campania “Luigi Vanvitelli”, 80138 Naples, Italy; delucaciro88@gmail.com (C.D.L.); virtuosoassunta@gmail.com (A.V.); 2 Department of Neurology, The Chaim Sheba Medical Center, Tel Hashomer, 52621 Ramat Gan, Israel; nicmaggio@gmail.com; 3 Department of Neurology and Neurosurgery, Sackler Faculty of Medicine and Sagol School of Neuroscience, Tel Aviv University, 6997801 Tel Aviv, Israel; 4 SYSBIO, Centre of Systems Biology, University of Milano-Bicocca, 20126 Milano, Italy

**Keywords:** coagulation, thrombin, proteinase activated receptors, activated protein C, amyotrophic lateral sclerosis, Alzheimer’s disease, Parkinson’s disease, multiple sclerosis, ischemic stroke, post-ischemic epilepsy, CNS cancer, addiction, mental health

## Abstract

Blood coagulation factors and other proteins, with modulatory effects or modulated by the coagulation cascade have been reported to affect the pathophysiology of the central nervous system (CNS). The protease-activated receptors (PARs) pathway can be considered the central hub of this regulatory network, mainly through thrombin or activated protein C (aPC). These proteins, in fact, showed peculiar properties, being able to interfere with synaptic homeostasis other than coagulation itself. These specific functions modulate neuronal networks, acting both on resident (neurons, astrocytes, and microglia) as well as circulating immune system cells and the extracellular matrix. The pleiotropy of these effects is produced through different receptors, expressed in various cell types, in a dose- and time-dependent pattern. We reviewed how these pathways may be involved in neurodegenerative diseases (amyotrophic lateral sclerosis, Alzheimer’s and Parkinson’s diseases), multiple sclerosis, ischemic stroke and post-ischemic epilepsy, CNS cancer, addiction, and mental health. These data open up a new path for the potential therapeutic use of the agonist/antagonist of these proteins in the management of several central nervous system diseases.

## 1. Introduction

The intricate and robust equilibrium between blood coagulation and bleeding is an essential process involving cellular and protein components, and evolved mainly to repair injured vessels and avoid acute bleeding [1].

The coagulation mechanism consists of three phases: initiation, amplification, and propagation, eventually followed by fibrinolysis, allowing the tissue to repair itself. The initiation is prompted by factor VII (FVII), which is the only human factor that circulates in both an inactive and an active (FVIIa) state to monitor the endothelium for sites of damage and tissue factor (TF) exposure. The TF–FVIIa complex activates Factor IX (FIX) to FIXa and Factor X (FX) to FXa. FXa indeed transforms a trace amount of the zymogen prothrombin in thrombin [2]. The TF–FVIIa-FXa complex can be rapidly inhibited by tissue factor inhibitor (TFPI). Antithrombin III is instead able to block FXa and thrombin. Traces of FXa and thrombin indeed provide a scouting signal that the tissue barrier is damaged, while TFPI and antithrombin III prevent amplification phase initiation without high levels of TF [3]. When the damaged tissue exposes enough TF, thrombin diffusion lead to platelet activation, membrane phospholipid exposition, and the release of granule contents. On the platelet surface thrombin cleaves Factor XI (FXI) to FXIa and Factor V (FV) to FVa and also cleaves von Willebrand factor (vWF) releasing Factor VIII (FVIII) [3].

Prothrombin, FVII, FIX, and FX are post-translationally modified by a vitamin K-catalyzed enzyme that converts glutamic acids to γ-carboxyglutamic acid (Gla) [4], doubling its negative charge. Thus, Gla residues ionically bind Ca^2+^ on the damaged phospholipidic layer to form protein complexes that favor and hasten clot formation but regulate it too, confining it to the damaged loci with limited dissemination [4]. Lack of vitamin K or its antagonism by dicoumarols inhibits Gla residues formation and slows down the coagulation cascade [4].

Thrombin consequently activates FVIII to FVIIIa [5] then FIXa and FVIIIa, bind to a plasma membrane (often offered by platelets), forming a so-called “tenase” complex. This complex is able to rapidly generate FXa, being composed by FIXa, FVIIIa, FX and Ca^2+^ [6] and is far more efficient than TF–FVIIa.

FXa begins assembling the prothrombinase complex composed of FVa, FXa and Ca^2+^ that ultimately converts prothrombin to thrombin with high efficiency. Thrombin cleaves fibrinogen to fibrin (the essential protein of the hemostatic clot), which is crosslinked and stabilized by the Factor XIII (FXIII) and can be disassembled by the protease plasmin (plasminogen activated form) [7].

FXIII, on the other hand, is a circulating tetramer consisting of two “A” subunits (FXIII A) carried by two “B” subunits (FXIII B). FXIII undergoes a thrombin-mediated cleavage activation of the FXIII A and requires Ca^2+^ as a cofactor to prompt FXIII B release to allow the catalytic site conformational opening, giving the transglutaminase-activated FXIII (FXIIIa) its pivotal role in the final fibrin cross-linking [7]. At the same time, on the intact wall vessels, thrombin inhibits itself through a feedback reaction: it binds to thrombomodulin, an endothelial transmembrane protein, and activates Protein C (activated Protein C, aPC). aPC, together with Protein S and TFPI, inactivates the coagulation FVa and FVIIIa and inhibits FXa, thereby quenching the coagulation process [8,9].

Moreover, thrombin interacts with its substrates depending on sodium cations. The sodium-bond “fast” form cleaves procoagulant substrates more efficiently, while the sodium-free “slow” form easily binds thrombomodulin [10,11]. Even if experimental data have been collected over the years, the definite structures and kinetic changes between these forms are still debated [10].

The circulating residues of thrombin are blocked by circulating serine protease inhibitors (serpins) such as antithrombin-III, TFPI, or heparin cofactor II, which can inhibit thrombin in the presence of dermatan sulfate or heparin; plasmin is inactivated by another protein of the same family, called α2-antiplasmin) [12,13].

Therefore, coagulation is modulated by feedback and feedforward mechanisms, which in physiologic conditions promote or prevent clot formation, differentiating injured from normal sites. Even if the cascade is constituted by several factors, we will focus on the final steps, which address the design of new therapies, i.e., the non-vitamin K antagonist oral anticoagulation (NOAC) approach adopts thrombin (dabigatran) or FXa (apixaban, rivaroxaban) inhibition [14].

However, recent studies have demonstrated that these proteins play a key role in the physiology of the cellular compartment, with a particular focus on the central nervous system (CNS) and consequently on its pathology [15,16,17,18,19].

**Figure 1:**
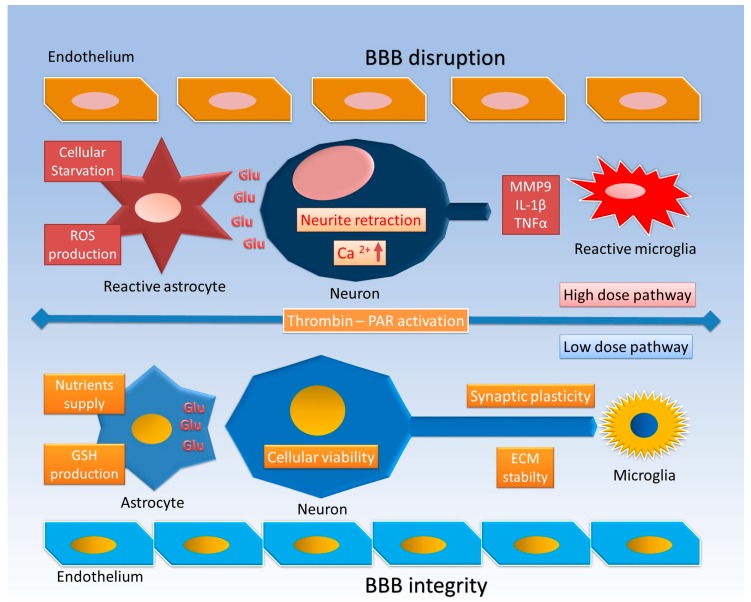
Thrombin effects at low and high doses. At low doses, astrocytes lose their star morphology maintain their fundamental role in nutrient supply, glutamate (Glu) sinking, and glutathione (GSH) production, thus promoting cell survival and synaptic plasticity; the blood–brain barrier (BBB) and extracellular matrix (ECM) both maintain their stability. At high doses, reactive astrocytes and microglia proliferate, lose their Glu regulatory function, and produce reactive oxygen species (ROS) and inflammatory cytokines (interleukin 1β (IL-1β), tumor necrosis factor α (TNFα)); neurons suffer neurite retraction, intracellular Ca^2+^ upregulation, and finally cell death, while the BBB is damaged and the ECM is digested by matrix metalloproteinases (MMPs).

The pathophysiology of thrombin in CNS cells, as shown in many experimental models, involves a wide spectrum of effects. It induces neurite retraction [20], morphological changes, and mitosis of astrocytes and/or microglia [21,22], and finally it affects cell viability [23] (Figure 1).

The effect of thrombin on astrocytes seems to be dose-related, showing different effects at low and high doses. At low doses it produces morphological changes with loss of canonical star morphology [24] and induction of thrombin inhibitors (protease nexin I, plasminogen activator inhibitor 1) and heat shock proteins (HSPs), and thus promotes cell survival [25,26]. At high doses it determines the astrocytic proliferative effect with inducible nitric oxide (NO) synthase (iNOS) [27], inflammatory cytokine production, and finally cell death [28]. These findings are consistent with “in vivo” effects [29] (Figure 1).

A low-dose preconditioning with thrombin has been found to reduce the inflammatory response and prevent neuronal death in both ischemic and hemorrhagic models [8,29]. These results may be a combination of direct thrombin action on neurons and indirect action on the other CNS cells and extracellular matrix (ECM) as components of the tetrapartite synapse [30,31,32,33].

**Figure 2:**
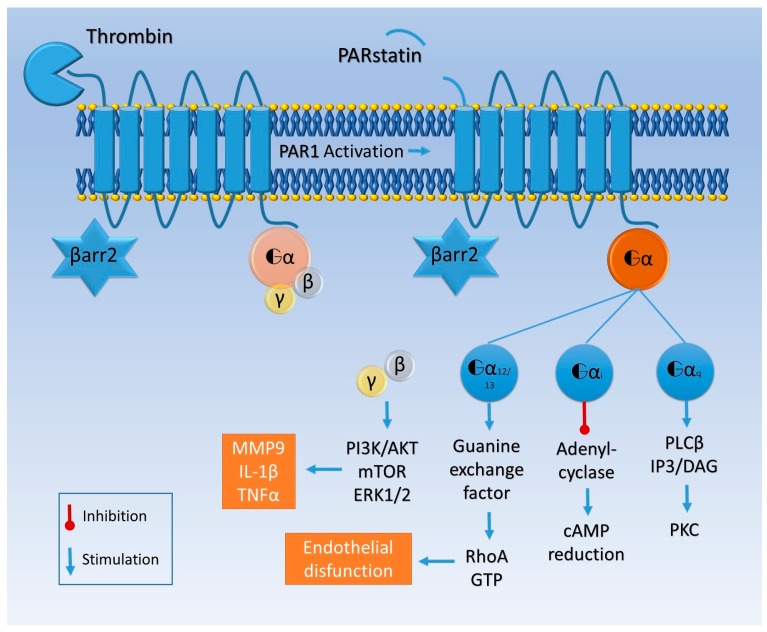
Thrombin cleaves the amino-terminal domain of PAR1 and triggers intracellular signaling. The residual 41 amino acid peptide is called parstatin. PAR1 can be associated with Gαq, Gαi/o, or Gα12/13 proteins. Gαq activates phospholipase C β (PLC β), which catalyzes the formation of inositol trisphosphate (IP3) and diacylglycerol (DAG), which in turn activates protein kinase C (PKC). Gαi blocks adenylyl cyclase and reduces cyclic adenosine monophosphate (cAMP). Gα12/13 is associated with guanine exchange factors and activates RhoA. βγ subunits activate the phosphatidylinositol 3 kinase (PI3K) pathway, leading to the production of MMP9, IL-1β, and TNFα. This is the “classic” PAR1 activation, and in this scenario β arrestin 2 (βarr2) remains inactive.

The majority of these changes have been related to proteinase-activated receptors (PARs), widely expressed on astrocytes, oligodendrocytes, neurons, and all the constituents of the vessel wall [34,35,36]. PARs are part of a transmembrane G protein receptor (GPCRs) family, referred to in other works as seven-transmembrane domain receptors (7TMRs), also expressed in brain tissue [37], whose activation is prompted by a serine protease-mediated proteolytic cleavage of the extracellular amino-terminal region (Figure 2) [38].

The PARs family, involved in hemostasis, phlogosis, cancer development, and embryologic differentiation, comprises PAR1, PAR2, PAR3, and PAR4 [39]. Thrombin, activated factor VII (FVIIa), and FXa are able to activate PARs on the cell surface of various tissues. Robust evidence states that thrombin can canonically activate, unmasking via cleavage their tethered ligand, PAR1, PAR3, and PAR4 [39]. Recently, however, it was shown that thrombin, at concentrations achievable at injury sites or in cancer pabulum, can also directly activate PAR2 [40]. Nonetheless, high concentrations of coagulation proteases could cleave PARs in vitro or in cellular models without physiological relevance, hence these data need to be interpreted and analyzed carefully [41].

Thrombin activates with higher potency PAR1 and PAR3 rather than PAR4 due to their hirudin-like motif [42]. Trypsin can activate PAR2 with nanomolar concentration [43] and has the same affinity of thrombin for PAR4. PAR3 seems incapable of acting as a receptor itself, due to a short cytoplasmic domain [44], but can work as a cofactor for other PARs, eventually increasing their affinity for thrombin [45] or causing their allosteric modulation [41]. Also PAR1, PAR2, and PAR4 can form heteromers with an even more complex variety of responses [46,47]. 

Dimerization, in fact, is just one type of non-canonical activation of PARs, and can also be triggered by synthetic peptides, biased agonism, or other proteases without tethered ligand unmasking, leading to distinct pathways [41].

PAR1 activated by thrombin interacts with different G proteins, activating Gαq/11, Gαi/o, or Gα12/13 and thus prompting mostly inflammatory responses [48,49] upregulating the expression of cytokines such as *interleukin 1β (IL-1β)* and *tumor necrosis factor α (TNFα)* or cell adhesion molecules (e.g., *selectins*, *intracellular adhesion molecule 1 (ICAM1*), and *vascular cell adhesion molecule 1 (VCAM1*)) [50,51]. 

Moreover, PAR1 and thrombomodulin compete for the same site to bind thrombin, which binds to thrombomodulin with a higher affinity than to PAR1 [52]. Otherwise, PAR4 thrombin-mediated activation activates Gαq/11, RhoA GTPase, p38, and extracellular signal regulated protein kinase-1 (ERK-1, i.e., p44-MAPK) and ERK2 (i.e., p42-MAPK) signaling [53]. Different from PAR1, a higher concentration of thrombin is needed, as mentioned above, to cleave PAR4 and induce an inflammatory response, particularly in endothelial cells [54].

A variety of intracellular transduction is, indeed, fundamental for the pleiotropic activity of these receptors [55]. 

Otherwise aPC binds its co-receptor, endothelial protein C receptor (EPCR), and together they engage PAR (Figure 2) [56]. EPCR–aPC–PAR complexes are linked to caveolin-rich membrane regions and favor cell viability pathways, decreasing cell death rate in inflammatory models [54]. Finally thrombin does not bind a co-receptor; it entirely cleaves PAR and induces its internalization, while aPC alternatively activates PAR by a partial cleavage and does not promote its degradation [57]. Thrombin induces the guanosine triphosphatase (GTPase) rat sarcoma protein (Ras)-related protein A (RhoA) activation, while GTPase Ras-related C3 botulinum toxin substrate 1 (Rac1) remains inactive; the opposite happens with aPC activation [57]. Rac1 is pivotal for endothelial stability, while RhoA leads to endothelial dysfunction [57].

The aPC-mediated Rac1 activation is β arrestin 2-related. Indeed, aPC works as a β arrestin “biased” agonist of PAR and does not activate G protein signaling [58]. The missing link between β arrestin and the GTPase activation seems to be produced by disheveled 2 (Dvl-2), usually associated with frizzled receptors (FZD) [58] (Figure 2).

PAR1-3-4 are efficiently activated by thrombin cleavage, while PAR2 is activated by trypsin or tryptase [59]. PAR1, detected in neuronal cells, astrocytes, and microglia, is highly expressed in the human cerebral cortex, striatum, and hippocampus [34]. It affects synaptic plasticity by enhancing the *N*-methyl-d-aspartate receptor (NMDAR) currents [60], playing a pivotal role in hippocampus-related learning activity and memory formation [61,62,63,64].

CNS diseases and neurological diseases have been associated with these proteins and their pathway abnormalities through evident vascular damage (e.g., stroke or vasculitis). Moreover, these factors have often been correlated with a variety of neurological and psychiatric symptoms with any evidence of vascular injury. Here we review the coagulation cascade proteins and their associated cellular receptors in chronic and acute diseases affecting the CNS.

## 2. Neurodegenerative Diseases

### 2.1. Amyotrophic Lateral Sclerosis

Amyotrophic lateral sclerosis (ALS) is a motor neuron degenerative disease characterized by progressive cell loss of the pyramidal tract throughout the motor cortex, brainstem, and spinal cord [65]. ALS is not a homogenous disease, accounting for different clinical presentation and ALS variants (e.g., primarily lateral sclerosis and progressive muscular atrophy), representing an insidious diagnostic challenge even with the newly revised diagnostic criteria [66]. Although the most prominent sign is progressive motor dysfunction, ALS can also occur with a syndromic motor and cognitive impairment, mostly associated with frontotemporal dementia (FTD) [67,68]. ALS usually begins with a few motor districts but inevitably progresses to complete paralysis and ultimately death. An estimated 10% of ALS patients have a causative genetic recognized mutation, called familial ALS (fALS). The most frequently mutated gene encodes for superoxide-dismutase 1 (SOD1) with at least one putative pathophysiological mechanism involving reactive oxygen species (ROS) production [69].

In the ALS model, relying on mutant SOD1 (*mSOD1*), which mimics the most frequent human fALS [70], it has been shown that aPC can reduce ROS production by interfering with *mSOD1* and is able to block redox sensitive transcription factor Sp1 nuclear translocation [71]. The selective inactivation of *mSOD1* in the endothelium does not moderate the disease progression, stressing the role of aPC expression in non-vascular cells. Thus a role for aPC is to function “in loco” by crossing the blood–brain barrier (BBB). Furthermore, it seems that to suppress the *mSOD1*, enzymatic activity of aPC is necessary (mainly PAR1 and PAR3 activation) but not aPC anticoagulant properties [71].

**Figure 3:**
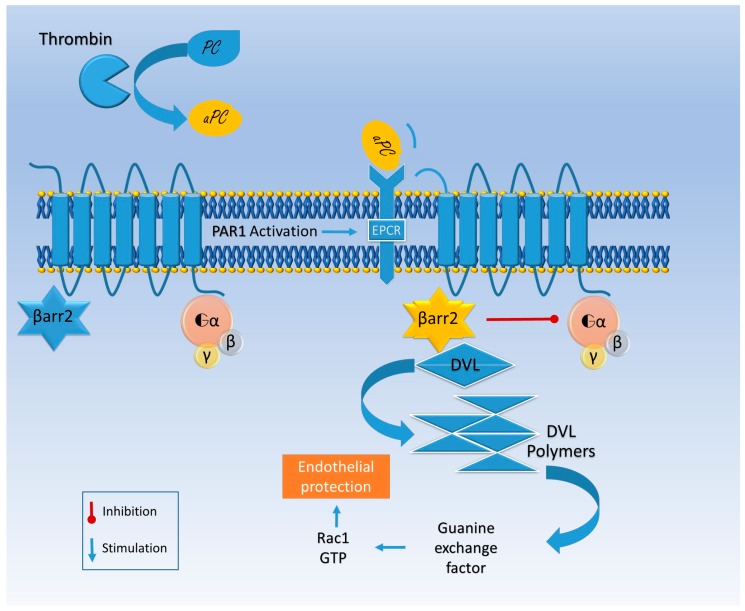
Representation of PAR1 activation by β arrestin 2 (βarr2)-biased pathway in endothelial cells. Thrombin activates protein C (aPC), a protease that binds endothelial protein C receptor (EPCR), promoting a different site of PAR1 proteolytic activation. In this conformation βarr2 is released from PAR1 and promotes disheveled (DVL) polymerization. This activity is associated with guanine exchange factor, which activates Rac1 (Ras-related C3 botulinum toxin substrate 1) and endothelial stability while the G protein signal is inhibited.

EPCR facilitates aPC transport through the BBB and seems to be essential for alternative PAR activation (the so-called β2arrestin bias) that guarantees endothelial integrity (Figure 3) [58,72], further supporting the hypothesis that EPCR’s impaired function is a putative player in the pathogenesis of neuronal degeneration.

Thrombin-mediated activation of PAR1, expressed on both neurons [73] and astrocytes [74], enhances excitatory synaptic transmission by an astrocyte-mediated glutamate release mechanism and exacerbates neuronal damage after brain injury [74]. Moreover, PAR1 increases l-glutamate spontaneous release onto substantia gelatinosa neurons from nerve terminals by a Ca^2+^-dependent process [75]. Whether upper motor neuron impairment provokes changes in glutamate metabolism or lower motor neuron damage initiates a degenerative process remains unclear. It seems that neuronal alterations cause abnormal glutamatergic activity, coupled with an excessive glutamate release in the motor cortex (Figure 1) [76]. Thrombin plays a key role in ALS pathogenesis, primarily through the modulation of interneuron calcium behavior and, among serine proteases, enhanced thrombospondin release. The following muscle accumulation constitutes an important biomarker of ALS progression [77,78].

Adverse events have been reported for the antiplatelet PAR1 inhibitor Vorapaxar (SCH 530348, Merck). In a clinical study TRA2P showed three cases of ALS and one of primary lateral sclerosis (PLS), mentioned as upper motor neuron lesion in the Vorapaxar-treated arm, against one case of ALS in the placebo group. In another study of the same molecule, TRACER, there was only one case of ALS registered in the placebo group. Finally, the total number of ALS cases in PAR1 inhibitor phase III studies was four versus two, comparing Vorapaxar with the placebo group. However, the expected number of ALS cases, considering the number of patients, the mean age of the cohorts, and the incidence of ALS, itself should be 2.8, which is almost in line with the observed data [79].

PARs are pivotal for various physiological functions and, certainly, their impairment is associated with a compromised intracellular and intercellular network, responsible for degenerative diseases. This process could potentially be a side effect of a drug specifically designed to inhibit platelets. Focusing on neurology, PAR1 and 2 are the most expressed in brain tissue, both on neurons and astrocytes regulating through thrombin-mediated cellular calcium influx: inflammatory response, pain susceptibility, tissue remodeling, and cell death [80,81]. However, the gathered data cannot rule out Vorapaxar being related to the reported ALS cases, because PARs are pivotal in impairing the synaptic transmission of spinal cord neurons [82] and off-target adverse effects altering nerve and muscle connectivity cannot be ruled out. Hopefully, next-generation PAR antagonists should be adequate to treat specific diseases and elucidate the pathogenesis of neuronal degenerative diseases. They could support a potential adverse effect produced by PAR antagonists on neuromuscular degeneration.

### 2.2. Alzheimer’s Disease

Alzheimer’s disease (AD) represents a progressive, degenerative, and nowadays irreversible pathology, mostly affecting cognition and leading to dementia [83]. The physicians Alois Alzheimer and Gaetano Perusini described AD in the first decade of the twentieth century [84]. In 2010 an estimated population of 35.6 million patients worldwide suffered from dementia and AD was responsible for 60% to 80% of these cases [85,86].

Anatomopathological lesions in brain tissues of AD patients are represented by amyloid β (Aβ) plaques, neurofibrillary tangles, neuron loss, and reactive gliosis [83,87].

A patient with classical AD experiences progressive and gradual loss of memory function with an amnestic syndrome defined as “hippocampal type”, with compromised episodic memory [88]. Although this is the most expressed phenotype, AD can present with variants (e.g., occipitotemporal, biparietal, logopenic) other than amnestic cognitive dysfunction, such as loss of visual identification, neglect, or limb apraxia [88]. This variety creates the necessity for biomarkers searched for in the cerebrospinal fluid (CSF) or through advanced neuroimaging. These markers are based on tau protein and Aβ to mirror, respectively, neurofibrillary tangles and amyloid deposition. There are also genetically determined forms of AD (with mutations or overexpression of *presenilin1-2* and *amyloid precursor protein*) or with high risk of AD development (*apolipoproteinE ε4* allel variant) [88].

Perusini in 1910 had already imagined a link between the vascular compartment and the “noxious agent” of AD: “Obviously, it remains an open question whether a noxious agent, which causes the whole disease, also acts on the blood vessels, or equally damages both the neuron and the blood vessels” [84].

Intravascular fibrin deposition in large vessels or capillaries can eventually reduce cerebral blood flow, resulting in chronic vascular occlusion and hypoperfusion, clearly demonstrated in AD patients [89]. Extravascular deposition, on the other hand, could sustain the inflammatory state existing in the brain parenchyma, characterized by endothelial tight junctions loosening, cell recruitment, and extracellular matrix remodeling [90]. Nonetheless, Aβ induces vascular matrix metalloproteinase (MMP-9) expression and may be significant in the etiology of spontaneous intracerebral hemorrhage (ICH) or cerebral amyloid angiopathy (CAA) [91].

FXIIIa and thrombin are also found in the Aβ depots in CAA, suggesting its local activation in vessel walls [92]. Moreover, FXIIIa forms stable complexes with Aβ1-40 and Aβ1-42 in a Ca^2+^-dependent manner that could not be inhibited by the FXIIIa active blockage site. The FXIIIa–Aβ aggregate formation is indeed independent of the cross-linking activity and seems to play a role in Aβ cytotoxicity and deposition in CNS blood vessel walls [92]. Thrombin instead can exert a protective or toxic role, depending on the protein concentration. A low level protects either neurons or astrocytes from apoptosis caused by various stressors [93], though high levels, as found in the microvasculature of AD patients [91] are neurotoxic and cause astrocyte death and interrupt the BBB, with concurrent edema and hemorrhage [94].

Aβ-42 has been reported as a prothrombotic factor, that can activate thrombin through FXII-mediated FXI activation, demonstrated by utilizing a wild-type Aβ-42 oligomer instead of Aβ-42 Dutch aggregates or oligomers [95].

Aβ-42 prothrombotic influence may be counterbalanced by the anticoagulant function of a soluble amyloid precursor protein (sAPP) that expresses an inhibitory domain for several coagulation factors, the Kunitz-type protease inhibitor (KPI) domain [96]. The sAPP is produced during the formation of Aβ-42, and thus the opposite action of the two proteins on the coagulation system may be in equilibrium. Accumulation in CNS parenchyma or blood vessels of these two fragments of the same precursor protein could be responsible for the patchy distribution of hemorrhage and thrombosis in the AD brain [97].

Aβ-42 oligomers definitively trigger a coagulation cascade in AD vessels, and their plasma levels are found to be significantly increased in both sporadic and familial AD at early stages of the disease or in prodromal mild cognitive impairment [98]. These processes, concurrent with other prothrombotic/hemorrhagic risk factors, usually coexisting in AD population, may explain the higher incidence of vascular events in these patients [99].

Eventually circulating Aβ-42 may induce factor consumption through FXII-mediated FXI activation, so there are higher levels of activated FXII (FXIIa) and lower levels of FXI and the complement factor C1 inhibitor (C1inh), which also inhibits activated FXI (FXIa), FXIIa, and Kallikreins (KLKs) involved in the coagulation intrinsic pathway [95].

Thrombin’s pathophysiological effects in AD seem to be mediated by the PAR-1/Gi/phosphatidylinositol 3-kinase (PI3K) signaling pathway [100]. Furthermore, thrombin has been shown to prompt MMP9 production with the exertion of pathological effects on the CNS cells and microenvironment [101] (Figure 1 and Figure 2). *PAR2* was also shown to be decreased in neurons of AD patients, while PAR2-activated glial cells enhance neurodegenerative processes [102].

Protease nexin 1 (PN1), produced by glial cells, is an endogenous CNS thrombin inhibitor; it has been shown to be extremely reduced in AD patients, along with a concomitant large increase of PN1–thrombin complexes. PN1 activity is increased in Aβ deposits, maybe as an effect of the aforementioned excess of thrombin [103,104].

### 2.3. Parkinson’s Disease

Parkinson’s disease (PD) is a neurodegenerative disease characterized by motor and non-motor signs, high variability of natural history, and pathologic expression (e.g., tremor dominant, postural instability and gait difficulty, young or late onset PD) [105]. The knowledge of the putative mechanisms that lead to a specific type of PD remains shallow, even though some differences in type-specific dopamine transporter (DAT) in vivo localization and functional network activation have been described [105].

PD can be defined, like other neurodegenerative disease, by its clinical, genetic, and pathological criteria. Clinical criteria, in particular for the diagnosis of probable PD, require the coexistence of bradykinesia and at least one among rigidity, 4 to 6 Hz resting tremors, and postural instability with at least three additional supportive criteria (e.g., response to therapy, unilateral onset, progressive disorder) [106]. The non-motor signs are mainly characterized by autonomic dysfunction, cognitive and behavioral disorders, or sleep and sensory (mainly olfactory) anomalies [106]. Pathological findings are based on cell loss and the presence of Lewy bodies, aggregates mainly composed of α-synuclein, mainly in the substantia nigra pars compacta [107]. Autosomal dominant, recessive, and X-linked genetic forms of PD have been found in the last two decades, with the first associated gene identified in 1997 as an *α-synuclein* missense mutation with autosomal dominant inheritance [105].

Given the pathogenic role of α-synuclein aggregates, studies have focused on its relation to PAR, particularly PAR1. Indeed, PAR1 inhibitors showed that α-synuclein upregulates MMPs, which increase the expression of IL-1β and TNFα (Figure 2) as well as NO and other ROS (Figure 1) [108]. Moreover, in a 4-phenyl-1,2,3,6-tetrahydro-pyridine (MPTP) mouse model, PAR1-deficient mice are protected; they show higher levels of dopamine and reduced microgliosis compared with wild-type mice [109].

Nonetheless, there is evidence that in PD, *PAR1* astrocytic expression is increased; at the same time, a low dose of thrombin seems to enhance the astrocytic protection of dopaminergic neurons through glutathione peroxidase production (Figure 1) [37]. 

Moreover, in PD what seems to be remarkable is the role played by PAR2 in long-term potentiation (LTP) and depression (LTD) phenomena [110]. This is mainly seen in cases characterized by dyskinesia, since anomalous LTP and LTD, along with dendritic spine density reduction, are supposed to be involved in determining iatrogenic dyskinesia [111]. Thus, it turns out that there is a remarkable therapeutic potential for PAR2 antagonism to reduce L-DOPA-induced dyskinesia and, presumably, positive psychiatric symptoms. Moreover, it will exert anti-inflammatory action with beneficial effects on neuronal cells’ vitality. The neuronal expression of PAR2 seems to behave as a proteinase regulator: acting by feedback circuitry, it will fine-tune synapse plasticity [112]. Evidence suggests that PAR2 is involved in LTP, LTD [110], and synaptogenesis, through the cleavage of the NR1 subunit of NMDAR, and has a role in neuronal activity modulation [113]. Notably, the only drug approved for dyskinesia is amantadine, whose pharmacodynamic property is supposed to be as an NR1 antagonist [114].

In a MPTP mouse model of PD, increased expression of PAR2 in the substantia nigra has been reported; furthermore, PAR2 inhibition reduced α-synuclein deposition and motor disability. PAR2 activation may prompt several intracellular transduction pathways, including mitogen-activated protein kinase (MAPK) and phospholipase C (PLC) signaling [115].

PLC signaling may lead to IκB kinase (IKK)α and IKKβ phosphorylation and nuclear factor (NF)-κB nuclear translocation [115]; this pathway, via the activated subunit p65, increases a specific DNA region’s histone acetylation. These effects determine the increased expression of specific genes [116] such as *α-synuclein* and the classic motor symptoms, at least in the MPTP animal model [117].

## 3. Multiple Sclerosis

Multiple sclerosis (MS) is defined as an idiopathic demyelinating inflammatory disease that affects the CNS, with the interaction between genetic predisposition and environmental factors not entirely clear [118,119]. Although the etiopathogenesis of MS remains undetermined, its major properties being phlogosis and related neurodegeneration, in recent years many steps forward have been made in understanding disease-modifying therapies [120].

Distinct phenotypes of this disease can be summarized as relapsing–remitting and progressive MS. Prominent inflammatory features with minor neuronal loss at the early stage of the disease are shown for the relapsing–remitting group, with both motor and non-motor symptoms (depending on the demyelinated CNS area) that partially resolve spontaneously or after therapy for every relapse [121]. A continuous neurodegeneration seems to be, instead, the paramount characteristic of the progressive MS form, with a dramatically lower rate of efficacy [121].

Inflammatory autoimmune diseases, like multiple sclerosis (MS), are characterized by an immunological switch. It activates the resident antigen-presenting cells, thus impairing the BBB and allowing for the margination and activation of specific T cells [122]. Depots of fibrin are constituent in the pathologic activation of the coagulative cascade during the CNS inflammatory process. They are non-diffusible and localized, thus may be the perfect candidate to mediate Th1 myelin-specific activation [122]. It has been shown that the appearance of fibrinogen in myelinated CNS zones correlates with autoimmune response and demyelination, T cell and macrophage induction via chemokine (C-X-C motif) ligand 10 (CXCL10) and chemokine (C-C motif) ligand 2 (CCL2), and Th1 differentiation through the upregulation of IL 12 [123]. In the experimental model of autoimmune encephalomyelitis (EAE), it has been demonstrated that the depletion of fibrinogen was efficient to inhibit T cell activation. Taken together, these findings show the key role of the coagulation process in inflammatory diseases and autoimmunity [124]. 

It is possible that fibrin acts as a prompting signal, enabling the antigen-presenting properties of resident cells, T cell localization, and memory of immune response [123].

Thrombin generated at the inflammatory loci can also increase platelet secretion, which in turn releases many factors favoring the aggregation or adherence to leukocytes, such as chemokines CCL3–5 and CXCL4–5 [125]. In addition, the ability of platelets to facilitate neutrophil adhesion was shown in a mouse model of autoimmune encephalomyelitis (EAE) [126].

Furthermore, mediators from platelet granules comprise nerve growth factors (i.e., brain-derived growth factor (BDNF)) and chemokines involved with MMPs in both hemostasis and inflammatory progression [127].

In the experimental EAE model the aPC has been shown to protect neurons. In detail, aPC anticoagulant and signaling activity were both required to ensure its function, as shown by using aPC analogues [128]. However, experiments have shown a different role for the endogenous aPC and the exogenous therapeutic molecule—specifically, the inhibition of the endogenous aPC in the same model improved the disease outcome [129]. This result was justified by the enhanced BBB permeability in endogenous aPC deficiency (Figure 3), followed by leukocyte infiltration, mainly the CD11b(+) subpopulation, known to be potent T cell suppressors, lowering the CD4(+) cells and reducing the burden of disease [129].

To identify the accurate time window related to the disease progression for the pleiotropic effect of aPC, the inhibition was shown to be effective only if the animals were treated before the disease onset [129]. This and other models [130] reveal the key role of the time window in the disease progression, besides the cellular (spatial) framework [131]. This evidence should suggest, in the design of clinical studies, that the timing of the therapy administration is as important as the right molecule choice.

Finally, in the MS patient brains, *PAR2* was increased in the glial cells in the white matter, while the neuronal expression was not significantly changed. Thus, the transduction system for the same receptor in glia plays a specific role [132].

## 4. Ischemic Stroke and Post-Ischemic Epilepsy

Stroke represents the most frequent (3–10%) event that cause seizures in elderly patients [133] [134]. In addition, post-ischemic seizures could prolong hospitalization, increase the mortality rate, and worsen postictal disability [135,136]. These factors enhance the already grievous social impact of stroke, one of the greatest among the Western diseases [137,138]. Recent guidelines have been established to manage strokes in general [139], but to achieve the same goal in post-stroke seizures further knowledge is required in this field [140,141].

One of the issues is that early damage in the ischemic area and eventually seizures onset are not assessable clinically, therefore the study of an animal model focusing in particular on coagulation protein may unravel some hypothesized mechanisms.

As previously discussed, the diverse effects produced by high and low doses of thrombin on the CNS revealed the beneficial outcome on synaptic plasticity produced by a low dose [60,142] versus the excitotoxicity and apoptosis caused by a high concentration [16,17,26,142]. Thrombin at high concentrations has been reported in peri-infarctual areas, supporting the hypothesis that it plays a crucial role in the recovery process in the ischemic penumbra. The study conducted on knockdown models of *PAR1* supports this assumption. It has been demonstrated that animals show better recovery and a smaller infarction area after the experimental ischemic stroke [143]. Pharmacological inhibition of the same receptor restores synaptic plasticity in a model of oxygen glucose deprivation (OGD). The hippocampal slices of *PAR1* knockdown animals, exposed to OGD, usually show normal synaptic transmission [144]. There is an opportunity to develop drugs to inhibit thrombin during cardioembolic strokes and following them to reduce the infarction area, via PAR1 inhibition. Thrombin is produced, moreover, in the brain and its levels alter the homeostatic behavior in both pathology and physiology [17]. In ischemic models such as OGD or transient medial cerebral artery occlusion (tMCAO), endogenous thrombin levels rise, which affects synaptic plasticity [16,144,145]. At the same time, FX levels increase too, suggesting a thrombin-mediated self-maintaining coagulation process following the stroke, contributing to the ischemic sequelae [146].

The high concentration of thrombin detected in the ischemic penumbra may also indicate the involvement of the endogenous thrombin, rather than the circulating one [147]. However, it is hard to determine if the brain-derived thrombin is primarily implicated in the short- or long-term reaction to the ischemic noxae.

Increased thrombin levels have also been associated with synaptic transmission changes, causing impaired excitability and likely post-ischemic seizures [17,19,61,64]. The latter has been hypothesized to result in impairment of the γ aminobutyric acid (GABA)-dependent inhibitory tone [61]. Furthermore, a lower concentration of thrombin has shown a modulatory effect on the synaptic excitability, increasing the paired pulse facilitation. PAR1 antagonist and diazepam administration counterbalanced this phenomenon [16].

The dose-dependent thrombin effects reported have been explained as a receptor sensitivity behavior theory. Essentially, different G proteins families may be more sensitive to a specific range of thrombin concentration, thus mediating different systemic effects [148]. These different thrombin-sensitive receptors may be located in different anatomical areas (i.e., the hippocampus), selectively regulating neuronal excitability [61]. Indeed, thrombin should be considered a putative trigger of post-ischemic epilepsy, affording the opportunity to inhibit PAR1/thrombin transduction (Figure 2) signaling to block seizures following cortical strokes.

Furthermore, a therapeutic approach targeting the serine protease aPC has the potential to improve stroke outcomes by interfering with the vascular and inflammatory changes that impair neuronal cells’ behavior [149]. The aPC network seems to act through the PAR1 mediated pathway, regulating the so-called neurovascular unit, thus favoring the stability of BBB and tempering the inflammatory response [149]. The aPC seems to enhance synaptic plasticity, improving recovery during the rehabilitation stage. It has been reported that the EPCR–PAR1–Sphingosine-1-phosphate receptor 1 (S1P1R) connections play a pivotal role in synapse adaptive plasticity (Figure 3) [62]. Whether neuronal or astrocytic PAR1 receptors are involved is still debated [62]. However, the co-expression of EPCR and PAR1 has been shown in the entire hippocampus. However, further studies are needed to shed light on the mechanism underlying this pathway. This will increase our knowledge concerning physiology and foster better therapeutic strategies [62].

## 5. Cancer

Astrocyte gliomas are the most frequent primary CNS tumors [150]. Gliomas are classified by the World Health Organization (WHO) classification of CNS tumors depending on their histology, molecular features, and biology, from grade I (low rate of growth, non-malignant, associated with long-term survival) to grade IV (high rate of growth, very aggressive malignant tumors) [150,151]. In adults, glioblastoma (GBM) is the most common CNS lethal form (grade IV WHO) with the hallmarks being diffuse necrosis, angiogenesis, uncontrolled proliferation, high infiltrative and metastatic power, resistance to apoptosis, and genetic instability [150,151]. In this tumor microenvironment, high thrombin levels are reached and there is often a coexistence of ischemic and bleeding areas, hence the relevance of coagulation factors to understand the development of the disease and eventually design specific drugs is paramount [40,152]. 

Thrombin, as mentioned above, sends its transduction signals through targeted plasma enzymes or PARs, thus affecting cell functions such as growth and viability. Thrombin can activate all PARs, with a lower affinity for PAR2, but it has been shown that the thrombin concentration in pathological conditions such as brain tumors or vascular injury is adequate to trigger the PAR2 signal downstream [40].

Tumor outgrowth is a mere error in cellular division and differentiation pathways, finally regulated at the proteomics level by MAPK, mainly ERK-1/2 [153,154]. Intracellular pathways include the PI3K pathway and PLC–PKC transduction (Figure 2); even if it is not a general rule, these cascades seems to be cell-type associated [155]. PAR1, however, is the major thrombin receptor, and its overexpression has been vastly studied in metastatic tumors, relating the PAR1 expression and the grade of tumor invasiveness [156]. Thus the PAR family nowadays represents one of the most interesting targets in the development of novel chemotherapy [157].

Moreover, PARs expressed on platelets seem to play a role in the spreading of tumor cells. Endothelial damage caused by tumor cells activates platelets that recognize sialated gangliosides on the lipid rafts of astroglial and neuronal cells surface, driving neuroinflammation in healthy cells [158], while tumor cells express many of the platelets receptors, a concept known as “platelet mimicry”. This phenomenon is combined with the property of tumor cells to secrete platelets agonists such as adenosine diphosphate (ADP) and thrombin, which cleaves platelets PAR1 and PAR4 receptor and activates mitogenic signals within the tumor microenvironment. Activated platelets secrete growth factors and proteinases that regulate tumor growth and invasion [159]. Furthermore, platelets can protect tumor cells by coating them and directly shielding them from physical stressors within the blood vessels or permitting evasion from the immune systems [160]. 

Tissue factor is upregulated in tumor cells like gliomas [152] as well as tumor-free tissue [161], producing thrombin and neurotoxic effects. At high thrombin levels, microglial activation has detrimental effects on neurons and astrocytes [162], characterized by edema formation and leukocyte infiltration [161]. Thrombin is involved in the production of nerve growth factor in gliomas and can stimulate vascular endothelial growth factor (VEGF) production, causing tumor angiogenesis [163]. Moreover, thrombin together with FXIII causes fibrin stabilization that supports tumor cells’ immunological masking [161]. KLKs, serine proteases implicated in a broad spectrum of physiological activities, including blood coagulation [164] and KLK6, KLK7, and KLK9 in particular, are associated with poor glioblastoma patient survival. KLK6 directly promotes glioma and astrocytoma cell survival, including resistance to radiation and temozolomide, in a PAR1-dependent manner [164,165].

Furthermore, in brain cancer thrombin may play a protective role; thrombin-processed *esophageal cancer-related gene 4 (Ecrg4)* has a chemotactic property for myeloid cells in vitro and in vivo. Ecrg4 mobilizes myeloid cells to mediate a pro-inflammatory role in suppressing tumor progression [166]. This unexpected mechanism may explain its downregulation in malignant gliomas [167].

## 6. Addiction

Drugs with psychoactive properties can act on the CNS and eventually result in addiction, an intricate disease with unbalanced neurotransmitters and receptor concentration primarily affecting the “reward system” [168]. Addiction could indeed be described as a self-sustaining state in which the capacity to control compulsive seeking is compromised, without thought to risk-taking or negative outcomes [169]. This condition can be treated and overcome but is usually difficult, and it can evolve into a chronic disease with devastating physical and social consequences. Actual treatments are limited and do not have a high long-term success rate [170], hence neurocoagulopathy may play a part.

Addiction can be considered a disorder of synaptic plasticity; factors associated with the coagulation cascade seem to be involved in these mechanisms. 

Amphetamines are known to activate MAPKs, the major regulators of endothelial tissue factor expression in neuronal cells [171]. Nicotine addiction induces a long-lasting effect due to the demethylation of the *monoamine oxidase-B (MAOB)* gene promoter, which results in a persistently high concentration of platelet MAO-B in former smokers who quit smoking an average of 13 years ago [172].

Evidence on opiate addicts confirms the occurrence of an impairment of the blood coagulation mechanism parallel to an increased plasma level of α2-macroglobulin and fibrinolysis [173]. Drugs of abuse, including morphine, methamphetamine (METH), and nicotine, increase the amount of dopamine released in the nucleus accumbens (NAc). These drugs activate post-synaptic dopamine (DA) receptors, namely D1 receptors (D1R). Activation of the D1R–cyclic adenosine monophosphate (cAMP)-protein kinase A (PKA) pathway leads to an increase in extracellular tissue-type plasminogen activator (tPA) activity in the NAc. The tPA-plasmin system regulates drug-induced DA release through activation PAR1, expressed on DAergic neurons, and is thereby involved in the reward effect/dependence [174,175]. 

Ethanol consumption and withdrawal also elevate tPA activity. In this scenario, tPA does not appear to function through plasminogen activation but through direct interaction with the NR2B subunit of the NMDAR in a non-proteolytic manner. Ethanol inhibits NMDARs activity, and NMDARs expression increases as an adaptive response. The rapid removal of ethanol relieves this inhibition on the expanded population of NMDARs, and when tPA acts on this large number of NR2B-containing NMDARs, it can prompt hyperexcitation and seizures [175,176].

## 7. Mental Health

The WHO defines mental health as “a state of well-being in which every individual realizes his or her own potential, can cope with the normal stresses of life, can work productively and fruitfully, and is able to make a contribution to her or his community” [177]. Diseases such as unipolar depression, bipolar disorder, or schizophrenia radically disrupt mental health.

Depression’s incidence in the general population is 5%; following heart attacks it rises to 20% [178] and conversely, depression is an independent risk factor for cardiovascular disease [179,180]. The mechanism by which these factors are related is not clear, but the relation seems to be supported bilaterally. Among others, scarce therapeutic compliance, voluptuary habits like smoking and alcohol consumption, and physical inactivity are suspected [181,182].

Nonetheless, a fascinating link resides in platelets, considering that these cells are the main sink of body serotonin, a pivotal substance in depression and platelet hyperactivity that could be linked to cardiovascular morbidity: platelet malfunction could connect depression and coagulopathy [182,183].

When matched with controls, the depressed population exhibits higher platelet activity, with increased expression of glycoprotein IIb/IIIa and P selectin [184].

Platelet granules also contain BDNF, which has been found to be decreased in depressed patient, with subsequent atrophy of limbic structures [185,186]. After treatment of depression with Citalopram, BDNF levels normalize to the level of controls, while single nucleotide polymorphism (SNP) in the *BDNF* gene has been found to be associated with depression and bipolar disorder [187]. The same SNP has been found with higher expression in patients with coronary disease and depression comorbidity [188]. Furthermore, the response of platelets to thrombin was greater for manic subjects than for depressed and schizophrenic patients through a differently modulated PKC signaling pathway [189].

Even if the findings described above are intriguing, a causal link between impaired mental health and platelets activation has not yet been confirmed.

Moreover, psychiatric patients show an increased procoagulatory state and more circulating fibrinolysis remnants compared to controls, though this can be partially reversed through appropriate therapy [190,191]

## 8. Conclusions

Robust experimental evidence proves a coagulation protein-mediated signal transduction throughout the CNS. The outlined endogenous versus circulating derived proteins theory needs further studies to assess whether there is still a reason to consider the two compartments separately as functionally divided in pathological conditions, preserving the BBB’s partial integrity. Conceptually, in fact, there is an extreme variability of protein interference and, consequently, drugs must be designed to tame the burden of disease where these mechanisms are malfunctioning. The PARs/thrombin interaction, with a clearly dose-dependent action, taken together with the temporal pleiotropy of aPC, nonetheless adds more complexity to future perspectives. There is still a need for additional data to shed light on the interaction between the cellular compartment (neurons, astrocytes, oligodendrocytes, and microglia) and the extracellular components of the tetrapartite synapse. The focus on the coagulation proteases seems to be promising for switching off the maladaptive plasticity occurring during CNS acute and chronic pathologies.
